# Hypocholesterolemia as a predictor of mortality in infective endocarditis

**DOI:** 10.1007/s11739-026-04449-0

**Published:** 2026-07-03

**Authors:** Fabian Patauner, Anna Maria Peluso, Iolanda Cafarella, Fabio Luciano, Raffaella Gallo, Rosa Zampino, Emanuele Durante-Mangoni, Lorenzo Bertolino

**Affiliations:** 1 Department of Precision Medicine, University of Campania ‘Luigi Vanvitelli’, Naples, Italy; 2 Department of Advanced Medical and Surgical Sciences, University of Campania ‘Luigi Vanvitelli’, Naples, Italy; 3 Unit of Internal Medicine and Transplants, A.O.R.N. Ospedali dei Colli—Ospedale Monaldi, Piazzale Ettore Ruggieri, 80131 Naples, Italy

**Keywords:** Total cholesterol, Infective endocarditis, Mortality, Hypocholesterolemia, Ischemic heart disease

## Abstract

Infective endocarditis (IE) is a thrombo-inflammatory disorder affecting the endocardium, showing a high morbidity and mortality. Circulating cholesterol levels generally decrease in severe infections but have not been analyzed in depth in IE. This was a single-center retrospective observational study including IE patients admitted between 2016 and 2023. After drawing ROC curves of lipid profile components for mortality, variables of interest were compared among two subgroups, determined by the best predictor’s cut-off value, using univariable and multivariable models. An adjusted logistic regression for in-hospital mortality was performed after inverse probability of treatment weighting. Survival analysis was carried out by the Kaplan–Meier method. Two-hundred-seventy-eight patients were included. Total cholesterol (TC) showed the highest AUROC for mortality (0.713 [0.625–0.801]). Patients with lower TC levels (≤ 134.5 mg/dL) were more likely to have ischemic heart disease (*p* = 0.026) and a higher creatinine (*p* = 0.006). Ischemic heart disease was an independent predictor of low TC levels (O.R. 2.190 [1.110–4.470]). The adjusted logistic regression showed a significantly higher risk of death during hospitalization in patients with lower TC levels (aOR 2.94, [1.80–4.92], *p* < 0.001). Survival analysis confirmed a higher in-hospital (*p* = 0.003) and 1-year (p < 0.001) mortality in patients with lower TC levels. Results of this study suggest that TC levels are strongly associated with in-hospital and 1-year mortality in IE. A cut-off for TC of 134.5 mg/dL was able to identify those at very low risk of death. Multicenter, prospective studies are needed to validate use of TC as a readily available and inexpensive marker of prognosis.

**Supplementary Information:**

The online version contains supplementary material available at https://doi.org/10.1007/s11739-026-04449-0.

## Introduction

Infective endocarditis (IE) is a thrombo-inflammatory disease affecting the endocardium, mostly due to Gram-positive cocci, and characterized by high morbidity and mortality [1]. In-hospital mortality averages at about 20% of cases, although it varies in relation to microbial etiology and type of valve involved [2]. Age-standardized mortality rate is 0.9/100,000 person-years [3]. Older age, prosthetic valve involvement, hemodialysis, frailty, heart failure, cerebrovascular complications, septic shock, *Staphylococcus aureus* etiology, and periannular complications have been consistently identified as main risk factors for mortality [2].

As for other infections, several metabolic and immuno-inflammatory domains may be profoundly deranged in IE, including lipid metabolism. Generally, the greater the inflammatory response, the higher the impact on lipid metabolism [4].

Modifications of lipoprotein levels have been well described in several infectious syndromes, such as pneumonia and urinary tract infections, as well as in sepsis and septic shock [5–7]. In the acute phase, these conditions may be characterized by a reduction of total cholesterol (TC), low-density lipoprotein (LDL) and high-density lipoprotein (HDL) cholesterol levels. Also, increased triglyceride levels are commonly observed [8]. In these clinical settings, hypolipidemia was shown to be a strong predictor of both in-hospital and long-term mortality [5, 7]. Much less is known regarding lipid changes in patients with IE. This is particularly important in light of the close relationship existing between vascular and endocardial damage and IE vegetation development [9].

We previously showed that low HDL-C levels were independently associated with embolic complications and mortality in IE [10]. In a subsequent study, we found low levels of circulating small dense LDL particles correlated with unfavorable clinical outcomes [11]. Both lipid changes are associated with endothelial dysfunction and vascular damage. These observations suggest that lipoproteins may constitute one link between vascular damage, vegetation formation, and embolic complications, with possible important pathophysiological as well as prognostic implications in IE.

In this study, we tried to expand our assessment of the effects of lipid changes in IE, with the aim to further understand whether the lipid profile can serve in clinical practice as a predictor of a worse outcome. More specifically, we designed our study to test the hypothesis that patients with lower cholesterol fractions’ levels are at higher risk of in-hospital mortality.

## Methods

### Study design

This was a single-center, retrospective analysis on clinical and biochemical data obtained in the context of a prospective observational study (PIEPRO-*p*rospective *i*nfective *e*ndocarditis *pro*gnosis study) in place at our center (Monaldi Hospital, AORN dei Colli, Naples, Italy). Included were all patients ≥ 18 years admitted to our hospital between January 2016 and October 2023 for suspected IE and for whom the ESC diagnostic criteria [2, 12] for definite IE were met. We included both left- and right-sided IE cases, as well as native or prosthetic valve and cardiac implantable electronic device (CIED) related IE.

Since there is no standardized protocol in our Hospital regarding the admission blood tests and considering that not all patients were initially admitted to our own unit, not all had a complete lipid profile test done. Whether patients had their lipid profile tested on admission was mainly determined by chance. Accordingly, we excluded patients with missing initial lipid profile.

Data were collected filling in a paper case report form from the hospital medical records and electronic charts. Collected patient data included: demographic, blood chemistry and microbiological parameters, and diagnostic investigation results referring to the time of hospital admission; we also collected information on any surgical procedure and on in-hospital outcomes.

First, we evaluated whether lipid profiles correlated with mortality and which among TC, LDL-C, and HDL-C had the strongest ability to predict mortality, evaluating their area under the Receiver-Operating Characteristic (ROC) curve. Second, the strongest variable was used to divide our cohort into two groups according to an established best cut-off obtained from the ROC curve by the Youden index. Study variables were compared between the two groups by univariable and multivariable analyses. In addition, an adjusted logistic regression was performed to reassess mortality after adjustment with inverse probability of treatment weighting (IPTW) for variables which: i) differed between the two groups for a standardized mean difference (SMD) ≥ 0.10 and/or ii) are established risk factors for mortality in IE.. Finally, a survival analysis of the two groups was carried out by the Kaplan–Meier method.

This study conformed to STROBE guidelines for reporting observational studies [13] and was approved by the Ethics Committee of our institution (prot. N. AOC 11110/2020). Written informed consent was obtained from each patient.

### Analyzed variables

Collected patients’ data included: sex, weight, age, presence of prosthetic material, history of ischemic heart disease, peripheral arterial disease, cerebrovascular disease, diabetes mellitus, liver cirrhosis, and antiplatelet and/or anticoagulant drug use. Among blood tests, lipid profile, C-reactive protein (CRP), neutrophil count, and creatinine levels were included. Data regarding microbiological test results, such as blood or valve cultures, imaging tests assessing valve vegetation dimensions or embolic events, and cardiac surgery information were collected as well. In-hospital mortality was defined as the occurrence of death for any cause during hospital stay for IE.

### Statistical analysis

Categorical and nominal data were expressed as numbers and percentages, while numerical data were expressed as mean and standard deviation (SD) or as median with interquartile range (IQR).

The value with the highest Youden index obtained after drawing an ROC curve for in-hospital mortality defined the cut-off which was used to distinguish the two study subgroups.

Fisher’s exact test or Chi-square test were used to compare differences for nominal variables. Numerical variables were compared with nonparametric Mann–Whitney’s *U* test or Kruskal–Wallis test.

A *p* value < 0.05 identified variables considered significantly associated with outcomes of interest. All variables significantly associated to the outcome were included with a stepwise backward conditional analysis in a multivariable logistic regression model to identify independent predictors of the outcome. Missing data were excluded from analysis. After IPTW adjustment based on different variables as specified above, an adjusted logistic regression was performed to reassess mortality. Kaplan–Meier curves for mortality were drawn in the two study subgroups and differential survival was analyzed with log-rank test. All statistical tests were computed using SPSS v. 29 (Armonk, NY: IBM Corp).

### Sample size calculation

Based on previous studies [10, 11], we expected a 15% mortality rate difference between the two study groups defined according to the cut-off cholesterol level. Accordingly, to obtain a power of 80% and an *α* = 0.05, a sample size of 240 patients was needed with at least 120 patients in each group.

## Results

Between January 2016 and October 2023, 326 patients were admitted to our hospital with a diagnosis of definite IE. Of them, 278 (85.3%) had lipid profile tested on admission and were included in this analysis (Supplementary Fig. 1).

Clinical features are shown in Table 1. Most patients were men (68.3%) and the median age was 66 years. Considering comorbidities, diabetes mellitus and history of ischemic heart disease were found in 23.7% and 24.8% of patients, respectively. Regarding IE features, prosthetic valve IE was diagnosed in almost one-third of patients (35.3%) and *Staphylococcus aureus* etiology was present in 18.7%. One-hundred-sixty-three patients (58.6%) underwent cardiac surgery during the same hospitalization.

**Table 1:** Clinical features of the 278 IE patients studied

Parameter	
Sex,* male*	190 (68.3)
Weight, *kg*	73 [65–82]
Ethnicity, *Caucasian*	277 (99.6)
Age, *years*	66 [56–73]
Smoking history, *yes*	151 (55.1)
Prosthetic valve IE	98 (35.3)
CIED IE	44 (15.8)
Embolism	92 (33.1)
Ischemic heart disease	69 (24.8)
Peripheral arterial disease	36 (12.9)
Cerebrovascular disease	32 (11.5)
Diabetes mellitus	66 (23.7)
Liver cirrhosis	5 (1.8)
Chronic kidney disease	53 (19.1)
*S. aureus* etiology	52 (18.7)
Cardiac surgery	163 (58.6)
Antiplatelet drugs	98 (35.3)
Anticoagulant drugs	112 (40.2)
C-reactive protein, *mg/dl*	7.1 [3.77–13.72]
Vegetation size, *mm*	14 [9–20]
Creatinine, *mg/dl*	1.1 [0.80–1.82]
Neutrophils, *cells/μL*	7781 [5445–11897]
Total cholesterol, *mg/dl*	126 [101.7–160.2]
LDL-C, *mg/dl*	70.4 [52.0–95.9]
HDL-C, *mg/dl*	29.0 [20.2–37.0]
Triglycerides, *mg/dl*	128.5 [93.7–166.2]

Data are *N* (%) or median [IQR]

*CIED* cardiac implantable electronic device, *IE* infective endocarditis, *LDL-C* low-density lipoprotein, *HDL-C* high-density lipoprotein

Median levels of TC, LDL-C, HDL-C, and triglycerides were 126 mg/dL, 70.4 mg/dL, 29.0 mg/dL, and 128.5 mg/dL, respectively.

**Fig. 1:**
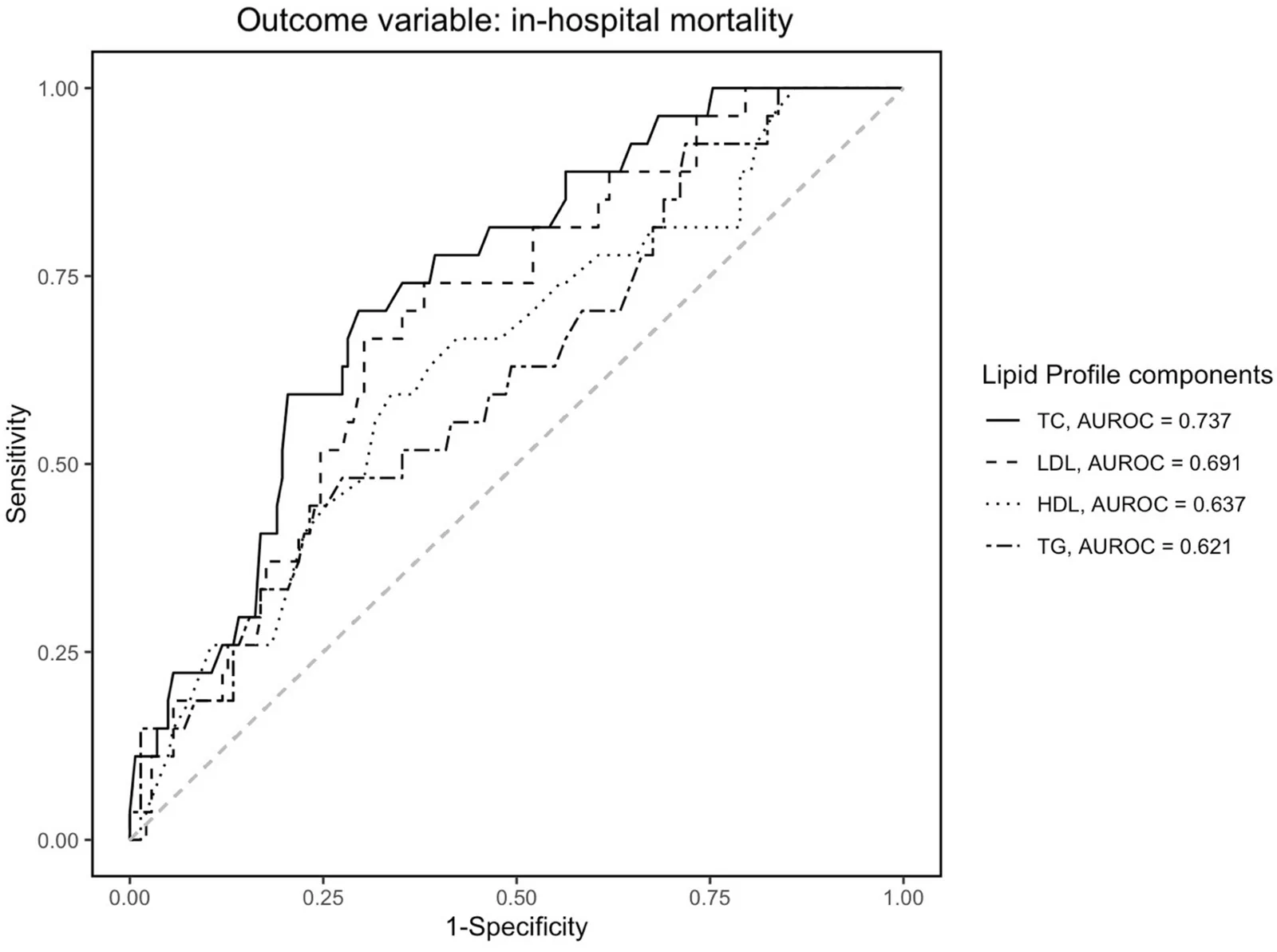
Receiver-operating characteristic (ROC) curves of lipid profile components for in-hospital mortality. *HDL* high-density lipoprotein, *LDL* low-density lipoprotein, *TC* total cholesterol, *TG* triglycerides

After ROC curve analyses, TC resulted the best predictor of hospital mortality, with a cut-off value equal to 134.5 mg/dL showing a specificity of 51% and a sensitivity of 79%, respectively (AUROC: 0.737, 95% CI 0.644—0.831; *p* < 0.001) (Fig. 1). Correspondingly, negative and positive predictive values were 92% and 25%, respectively.

Subsequently, the 278-patient cohort was divided into 2 subgroups based on the identified cut-off and further analyses were performed, see Table 2. At univariable analysis, patients with TC values ≤ 134.5 mg/dL had worse renal function (creatinine 1.2 vs 1 mg/dL, *p* = 0.006) and higher prevalence of prior ischemic heart disease (30.9% vs 18.6%, *p* = 0.026) and chronic kidney disease (24.8% vs 11.5%, *p* = 0.013). Additionally, a trend was observed for higher levels of CRP (7.8 vs 6.9, *p* = 0.061). Patients with TC levels ≤ 134.5 mg/dL were numerically more likely to be affected by liver cirrhosis (3.3% vs 0.0%, *p* = 0.063) and diabetes mellitus (27.5% vs 19.4%, *p* = 0.105). Since LDL-C, HDL-C, and triglycerides all showed correlation with TC levels (Supplementary Fig. 2), these were not included in the multivariable analysis.

**Table 2:** Univariable and multivariable analyses of factors possibly affecting total cholesterol levels in IE

Parameter	Total cholesterol levels ≤ 134.5 mg/dl, (*n* = 149)	Total cholesterol levels > 134.5 mg/dl, (*n* = 129)	*p* value	O.R. [95% C.I.]	*p* value
Sex,* male*	103 (69.12)	87 (67.4)	0.763		
Weight, *kg*	73 [65–84]	72.6 [65–80]	0.725		
Ethnicity, *Caucasian*	149 (100)	128 (99.2)	0.942		
Age, *years*	68 [56–75]	64 [55.0–72.5]	0.170	0.998 [0.982–1.015]	0.858
Smoking history, *yes*	81 (54.4)	70 (54.2)	0.992		
Prosthetic valve IE	54 (36.2)	44 (34.1)	0.710		
CIED IE	30 (20.1)	14 (10.9)	0.051		
Embolism	46 (30.9)	46 (35.6)	0.466		
Ischemic heart disease	45 (30.2)	24 (18.6)	**0.026**	1.773 [0.973–3.286]	0.064
PAD	18 (12.1)	18 (14.0)	0.688		
Cerebrovascular disease	17 (11.4)	15 (11.6)	0.931		
Diabetes mellitus	41 (27.5)	25 (19.4)	0.105	1.225 [0.664–2.274]	0.516
Liver cirrhosis	5 (3.3)	0 (0.0)	0.063		
Chronic kidney disease	37 (24.8)	16 (11.5)	**0.013**	2.190 [1.110–4.470]	**0.027**
*S. aureus* etiology	33 (22.1)	19 (14.7)	0.251		
Cardiac Surgery	82 (55.0)	81 (62.8)	0.182		
Antiplatelet drugs	55 (36.9)	43 (33.3)	0.565		
Anticoagulant drugs	63 (42.3)	49 (37.9)	0.514		
C-reactive protein, *mg/dl*	7.8 [4.05–15.90]	6.9 [3.25–11.4]	0.061	1.022 [0.995–1.052]	0.119
Vegetation size, *mm*	14 [10–20]	14 [8–20]	0.629		
Creatinine, *mg/dl*	1.2 [0.90–2.46]	1 [0.8–1.4]	**0.006**	0.970 [0.918–1.023]	0.259
Neutrophils, *cells/μL*	7750 [5175–12107]	7811 [5792–11595]	0.863		
LDL-C, *mg/dl*	54.6 [38.7–65.3]	96.8 [87.2–118.0]	< 0.001		
HDL-C, *mg/dl*	24.0 [19.0–31.0]	35.0 [27.0–43.2]	< 0.001		
Triglycerides, *mg/dl*	116.5 [89.7–151.2]	137.0 [106.2–180.0]	< 0.001		

Data are *N* (%) or median [IQR]

*CIED* cardiac implantable electronic device, *IE* infective endocarditis, *PAD* peripheral arterial disease

In bold are p values denoting statistical significance of the differences, as per the methods employed

As a result, logistic regression analysis indicated chronic kidney disease as the only independent predictor of a lower TC level (O.R. 2.190, 95% C.I. [1.110–4.470], *p* = 0.027), Table 2.

Next, an IPTW adjustment of TC levels for sex, age, smoking history, ethnicity, cardiac surgery, CRP, *S. aureus* etiology, diabetes mellitus, ischemic heart disease, and chronic kidney disease was performed, according to methodology described above (Supplementary Table 1). The absolute SMD of these variables before and after adjustment is shown in Supplementary Fig. 3. The resultant adjusted logistic regression for in-hospital mortality showed a significantly higher and independent risk of death in patients with lower TC levels (adjusted odds ratio 2.94, 95% C.I. 1.80–4.92, *p* < 0.001) (Table 3).

**Table 3:** Unadjusted and adjusted logistic regression for mortality according to total cholesterol levels

	Deceased (*n* = 48)	Discharged alive (*n* = 230)	OR [95% C.I.]	*p* value	aOR [95% C.I.]	*p* value
TC ≤ 134.5 mg/dl, *n* (%)	38 (79.1)	111 (48.2)	4.04 [1.99–8.92]	< 0.001	2.94 [1.80–4.92]	< 0.001
TC > 134.5 mg/dl, *n* (%)	10 (20.9)	119 (51.7)				

*TC* total cholesterol, *OR* odds ratio, *aOR* adjusted odds ratio

**Fig. 2:**
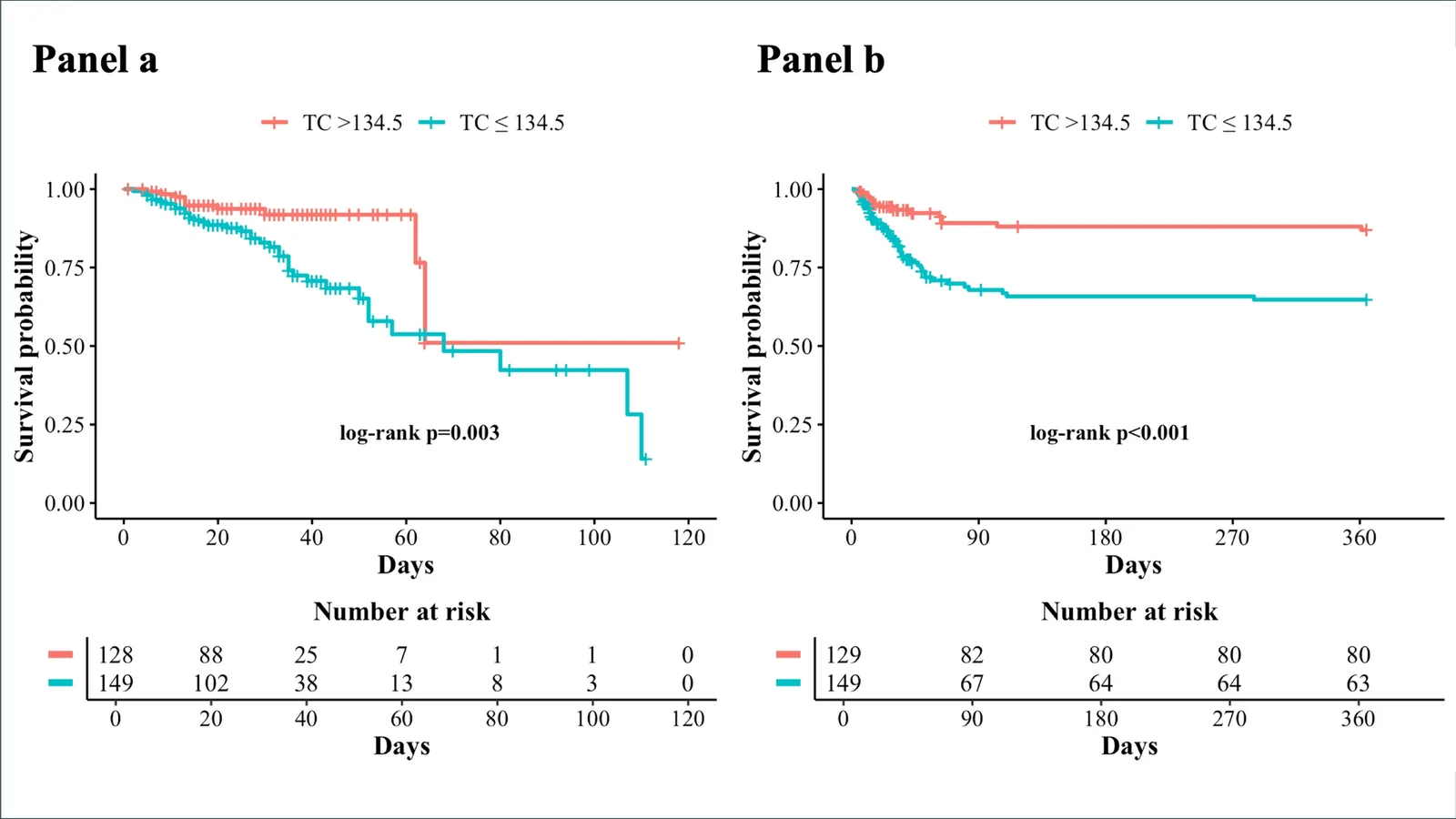
Panel a, Kaplan–Meier curves for in-hospital mortality. Panel b, Kaplan–Meier curves for 1-year mortality. In red patients with total cholesterol levels > 134.5 mg/dl. In light blue patients with total cholesterol levels ≤ 134.5 mg/dl. *TC* total cholesterol

We then assessed the possible association between baseline TC levels and other outcomes occurring during hospital stay. Lower TC levels were observed in patients developing de novo heart failure (103 vs 136 mg/dL; *p* = 0.015). In contrast, no association was found with embolic complications, including stroke, or with splenomegaly or use of cardiac surgery (data not shown). Survival analysis confirmed a significantly higher in-hospital and 1-year mortality in IE subjects with TC levels ≤ 134.5 mg/dL (log-rank *p* = 0.003 and *p* < 0.001, respectively; Fig. 2).

## Discussion

IE is a severe disease with significant mortality, making prognostic stratification an important goal to tailor the therapeutic approach. It is also a unique model of infection, where microbial factors interact with vascular damage and coagulation abnormalities in a complex pathophysiological interplay. This is why, we studied the prognostic implication of lipid abnormalities in IE, aiming in parallel to identify possible clues to the disease mechanisms.

Regarding mortality prediction, TC emerged as a strong and independent factor even after adjustment for major, potentially confounding covariates. Having a TC level > 134.5 mg/dL implied a very high likelihood (92%) of a favorable hospitalization outcome.

IE patients with more profound reduction of TC have both a higher rate of prior chronic kidney disease and a higher admission creatinine. Also, prior ischemic heart disease and liver cirrhosis were more prevalent among those with lower TC. These data are not surprising and provide interesting insights. Indeed, patients with chronic kidney disease experience profound lipid metabolic changes, leading to HDL-C reduction, alteration in LDL composition, and a net effect of TC reduction [14]. Although this condition may explain the higher creatinine levels, the latter could also be due to acute kidney injury in more severe IE presentations [15]. Moreover, patients with ischemic heart disease are frequently treated with cholesterol lowering agents [16, 17]. Liver disease affects lipid profile, such that the greater the liver dysfunction, the lower the TC levels [18]. Overall, inflammation could link these comorbidities with TC levels in IE. Indeed, IE patients showing lower TC levels also had a trend for higher CRP. Considering CRP is an established predictor of in-hospital mortality, our findings identify hypocholesterolemia and hyperinflammation as related markers of a more severe disease course in comorbid IE patients [19–22].

To date, a few studies have addressed the potential association of cholesterol levels with IE prognosis. Our group previously reported how lower levels of HDL-C correlated with embolic events and predicted in-hospital mortality in a smaller cohort of IE patients [10]. Similar findings had been previously reported by Kahveci et al. [23]. We also studied the role of small dense LDLs, demonstrating their correlation with worse outcomes in IE patients [11]. No other studies specifically addressed the role of cholesterol as predictor of outcome in the acute setting of IE. We believe that the current data build upon our previous findings and provides enough evidence to suggest TC can now be used in clinical practice to early and easily identify IE patients for whom a more aggressive or tailored approach should be employed.

Regarding IE disease mechanisms and pathophysiology, cholesterol might have an interesting role, binding vascular damage with innate immunity. Although cholesterol is a well-recognized marker and one of the main contributors to the atherosclerotic cardiovascular disease [24], its functions as a player in the innate immune response have been receiving less attention [25, 26].

Cholesterol-rich lipoproteins act at different levels of the innate immune response. Lipoproteins can act as a scavenger for bacterial exotoxins like lipoteichoic acid (LTA) and lipopolysaccharides (LPS) via a direct inclusion of the lipid component or through the lipopolysaccharide-binding protein [27–29], preventing an overregulated inflammatory response, mainly mediated by Toll-like receptors [8, 30, 31]. Moreover, cholesterol is a main component of lipid rafts, and HDL-C—influencing cellular signaling—is able to reduce proinflammatory cytokines, and attenuate type I interferon response to LPS [25]. In addition, HDL-C contributes to the integrity of vascular system carrying sphingosine-1-phosphate, enhancing endothelial adherent junctions [32]. Furthermore, a reduction of HDL-C was associated with increased platelet activation [9, 33], that is a critical step in IE pathophysiology.

Intense inflammation exerts its influence on lipid metabolism throughout several mechanisms that act on all TC components. Serum phospholipase A2 and serum amyloid protein A, both acute phase reactants, are involved in HDL-C catabolism [34, 35]. Their increasing levels during inflammation contribute to HDL-C serum clearance [36]. Modifications of cholesterol pathways may lead to a reduction of LDL synthesis in the liver and an increased formation of oxidized LDL, that are rapidly removed from bloodstream by macrophages [37]. Moreover, in vitro studies in human fibroblasts, endothelial cells, and liver cells have shown that TNF-alpha enhances LDL degradation [38]. The influence of cholesterol levels on severe infections is supported by the recent publication of a phase 2 clinical trial—showing promising results—that addressed whether directly increasing cholesterol levels may tackle the worse prognosis observed in sepsis [39].

In IE patients, no similar clinical trials were performed, but the evidence is somewhat conflicting. Indeed, two studies and one metaanalysis found the use of statins before IE onset to predict a better outcome [40–42]. In particular, Yu et al. showed that continuation of statin therapy during IE-related admission improved survival, and the higher the statin doses, the better the outcomes [41]. Although the positive effect on survival was deemed to be related to statin ‘pleiotropic effects’, an equally positive interaction with cholesterol metabolism cannot be ruled out. Indeed, in these studies, cholesterol levels of included patients were not available.

Beyond the potential effect on infection prognosis, our data also bind hypocholesterolemia with de novo heart failure in IE. This finding is particularly interesting, further alluding to a role of systemic inflammation as a driver of cardiac dysfunction in IE, as we previously suggested [43].

Reported studies, mainly performed in septic patients, suggest that cholesterol might be both cause and consequence of a dysregulated response, a severe infection, and a worse prognosis. Unfortunately, the design of our study is unable to provide direct evidence to establish whether lower levels of TC initiate or ensue from a more severe infective episode in IE.

On the other hand, our results suggest that the role of cholesterol in IE is worth to be addressed by further studies.

Limitations of this study are its retrospective nature the single-center design and the absence of detailed information regarding the chronic use of lipid lowering therapy. The adjusted analysis helped mitigate this potential bias. However, since the nutritional status of included patients, the spectrum of liver disease and IE presentation were not fully addressed in this study, the presence of residual confounding factors related to these conditions cannot be completely ruled out. In addition, by excluding patients with missing data for lipid profile, a potential selection bias may have occurred, although the lack of this evaluation upon admission seemed unrelated to differences in patient characteristics. Another limitation is the lack of external validation. Strengths of the study are its adequate sample size and the comprehensive adjustment for possible confounders.

In conclusion, the results of this study suggest that cholesterol levels are strongly associated with in-hospital mortality in IE. In our cohort, a cut-off value for TC of 134.5 mg/dL was able to identify those at very low risk of death. Further studies are needed to address mechanisms underlying the relationship of cholesterol levels with the pathophysiology of IE. Multicenter, prospective studies are needed to validate the identified cut-off and confirm the possible use of TC as a readily available and inexpensive marker of prognosis.

## Supplementary Information

Below is the link to the electronic supplementary material.

Supplementary file1 (PDF 485 KB)

## Data Availability

The dataset used for this article is available on reasonable request from the corresponding author.
