# Supplementary material for: Hypocholesterolemia as a predictor of mortality in infective endocarditis
Source: Intern Emerg Med. 2026 Jul 3;21(7):2599–607. doi: 10.1007/s11739-026-04449-0 (PMC13694383; doi:10.1007/s11739-026-04449-0)
Supplement: Supplementary file 1 [file 11739_2026_4449_MOESM1_ESM.pdf]

Supplementary Table 1. Standardized mean difference of study variables comparing patients according to TC level cut-off (134 mg/dl)

Variables	SMD
Sex	0.036
Ethnicity	0.125
Smoking history	0.121
Embolism	0.088
Ischemic heart disease	0.273
Diabetes mellitus	0.197
Cerebrovascular disease	0.011
S. aureus etiology	0.192
Antiplatelet drugs	0.070
Anticoagulant drugs	0.094
Cardiac surgery	0.175
Creatinine	0.026
C-reactive protein	0.182
Age	0.090
Prosthetic valve IE	0.045
CIED IE	0.259
Vegetation size	0.073
Weight	0.029
Neutrophils	0.111
Chronic kidney disease	0.323

CIED, cardiac implantable electronic device; IE, infective endocarditis; SMD, standardized mean difference

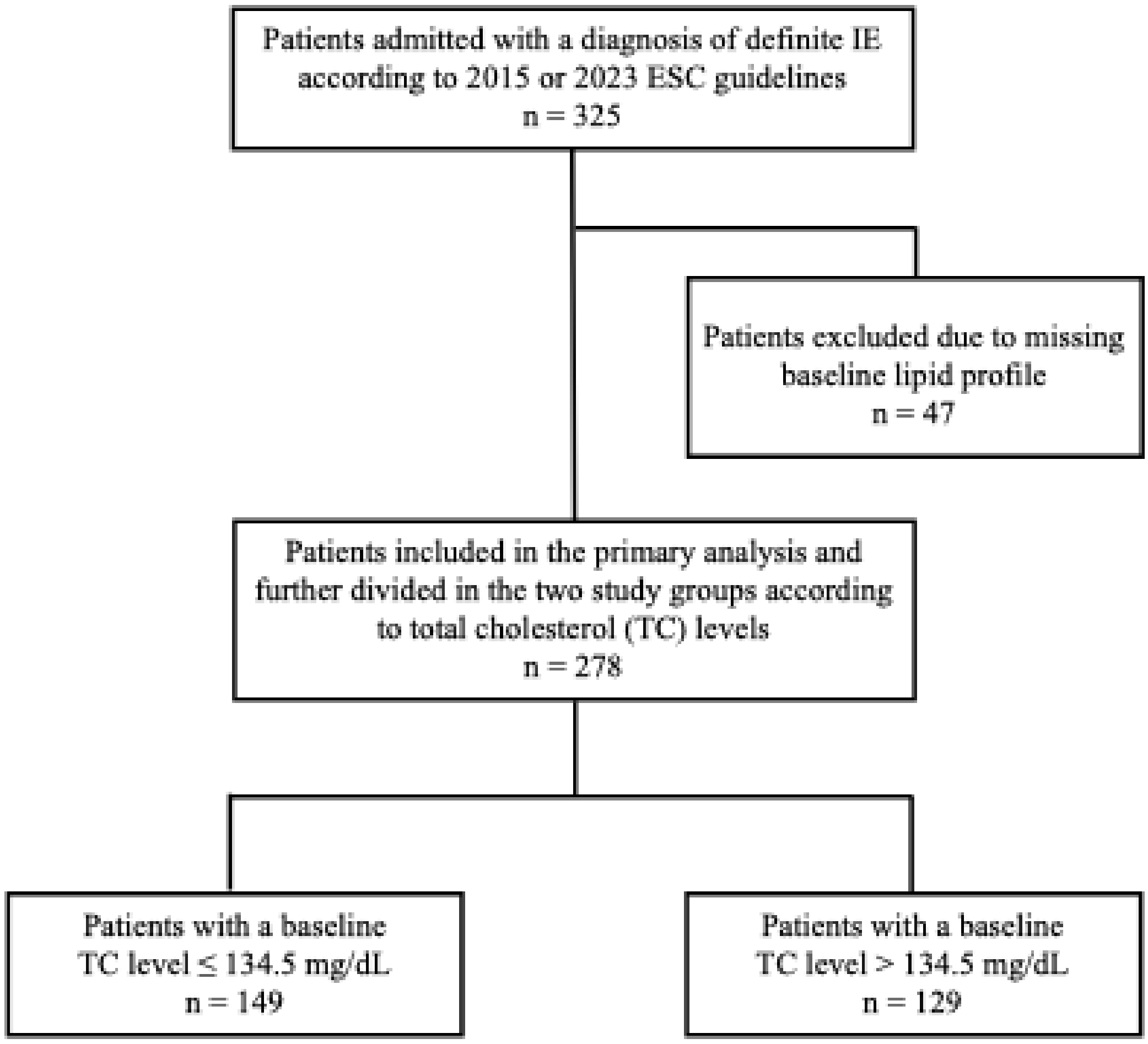

Supplementary Fig. 1. Study flow chart. IE, infective endocarditis; TC, total cholesterol

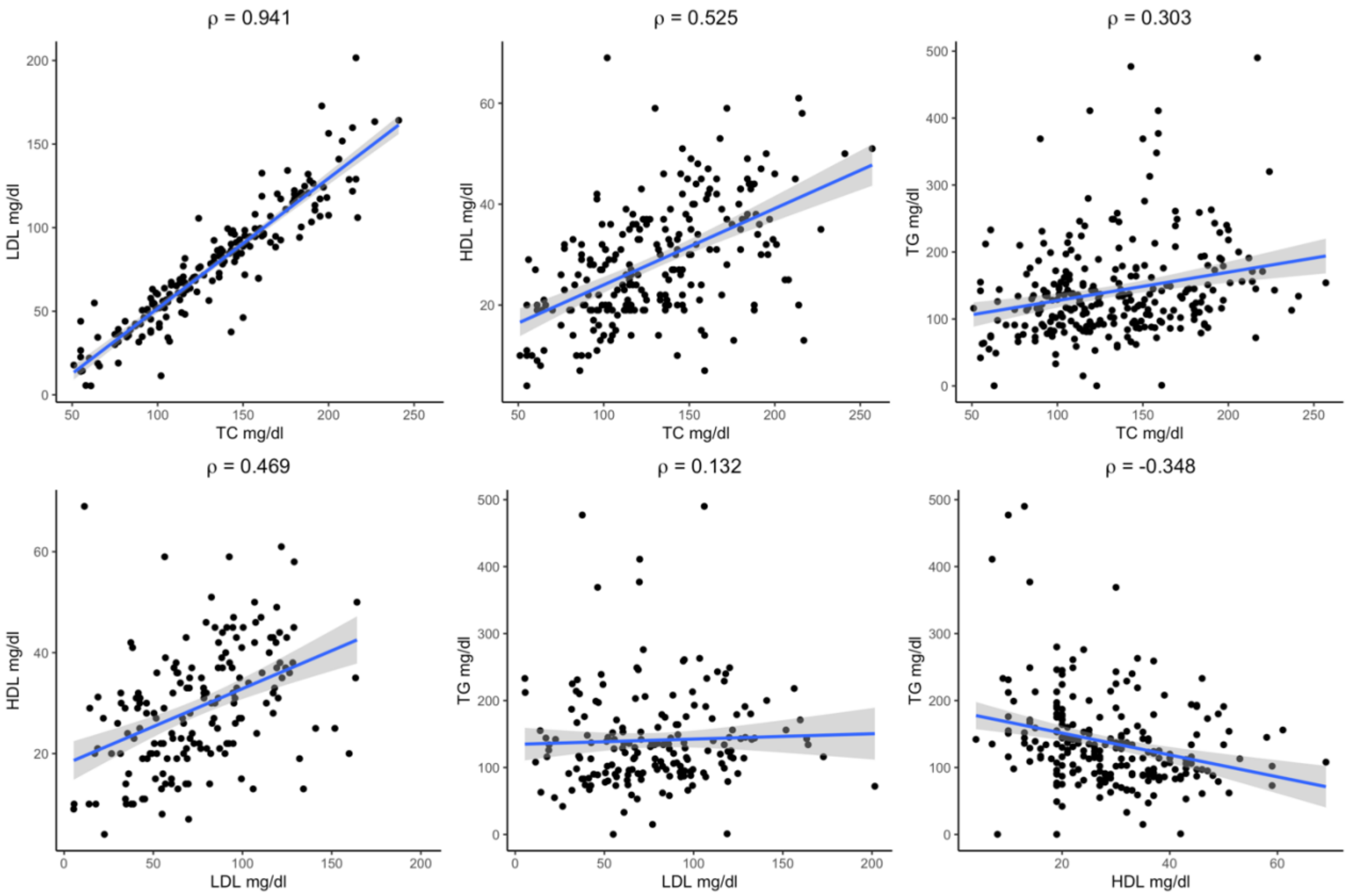

Supplementary Fig. 2. Spearman's correlation among lipid profile components. In blue linear regression line with its 95% C.I. in grey. HDL, high-density lipoprotein; LDL, low-density lipoprotein; TC, total cholesterol; TG, triglycerides.

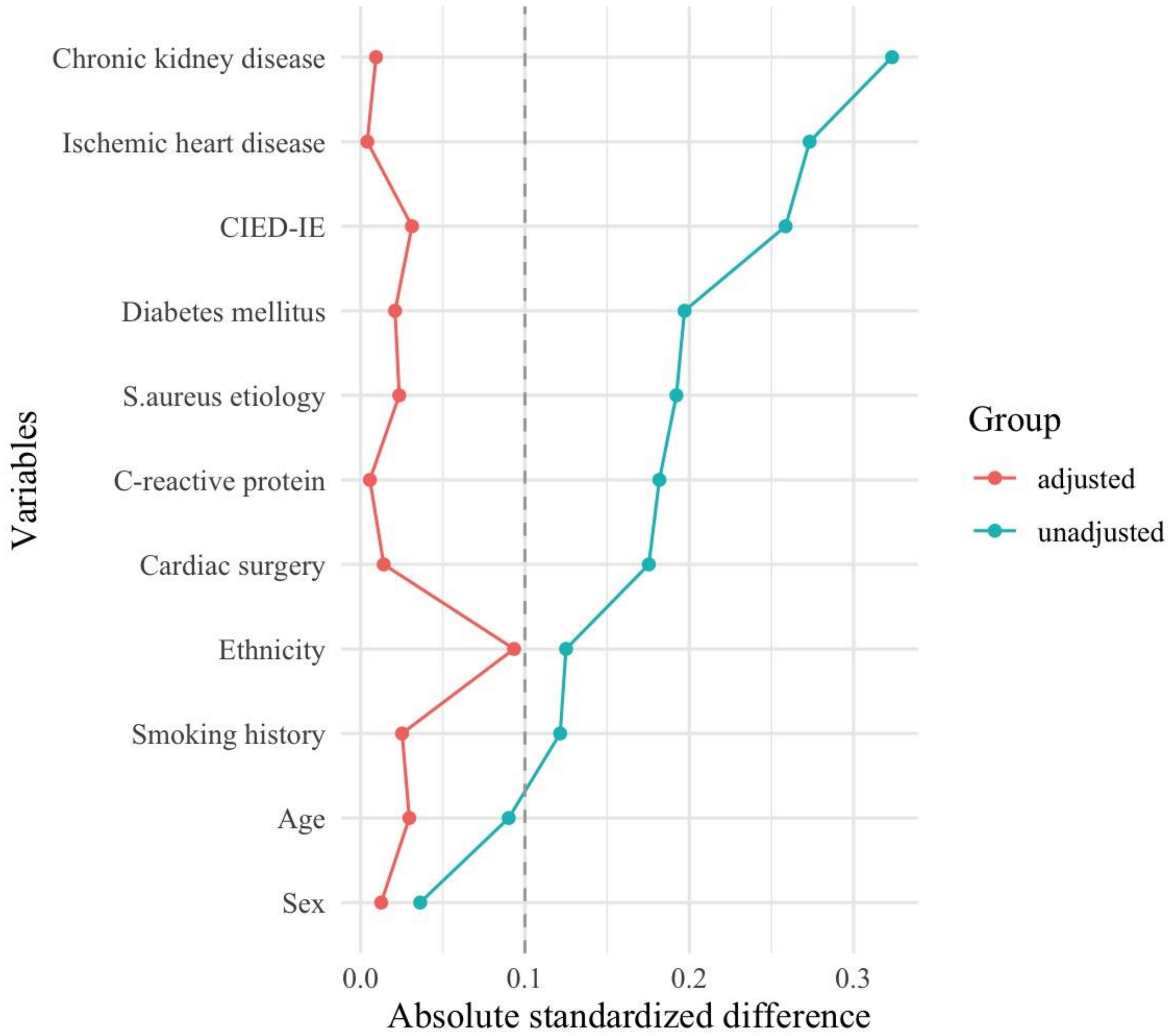

Supplementary Fig. 3. Standardized mean difference of variables compared in patients according to TC level cut-off (134 mg/dl) before (light blue) and after (red), inverse probability of treatment weighting (IPTW). CIED-IE, cardiac implantable electronic device-infective endocarditis
